# Ectopic Compound Odontoma in the Buccal Mucosa: Report of a Rare Case

**DOI:** 10.1155/2015/835171

**Published:** 2015-02-25

**Authors:** Aparna Venigalla, Leela Krishna Guttikonda, Hasini Nelakurthi, Suresh Babburi, Soujanya Pinisetti, Ajay Banerji Kotti, Lavanya Kalapala

**Affiliations:** ^1^Department of Oral Pathology, Drs Sudha & Nageswara Rao Siddhartha Institute of Dental Sciences, Chinoutpalli, Gannavaram, Krishna, Andhra Pradesh 521 286, India; ^2^Department of Oral & Maxillofacial Surgery, Drs Sudha & Nageswara Rao Siddhartha Institute of Dental Sciences, Gannavaram, Andhra Pradesh 521 286, India

## Abstract

Eruption of tooth into extraosseous locations is an extremely rare condition. We report a case of a six-year-old girl child with tooth-like structure erupting from the right buccal mucosa. Clinical, radiographic, and histopathologic examination suggested the diagnosis of compound odontoma. Very few cases have been reported so far, where tooth has been located completely in the soft tissue and a variety of names have been used for that condition. A brief review of the literature and the ambiguity in naming the situation is discussed.

## 1. Introduction

Odontoma is the most common benign odontogenic tumour showing slow-growth and nonaggressive behaviour [1, 2]. The term odontoma was coined by Broca in 1867 [3]. Odontomas are hamartomas composed of various dental tissues, that is, enamel, dentin, cementum, and sometimes pulp [2]. According to classification by World Health Organization (WHO), 1992, two types of odontoma have been recognized: (a) compound odontoma: malformations with the presence of all types of dental tissues and exhibiting an orderly distribution in the form of tooth-like structures; (b) complex odontoma: malformations in which all dental tissues are likewise represented but with a disorganized distribution [4–7]. Based on morphology, Garvey et al. in 1999 have further classified compound odontoma into three types: (a) denticular type which is composed of two or more separate denticles, each resembling a tooth; (b) particulate type which is composed of two or more separate masses of particles with dental tissues abnormally arranged; (c) denticuloparticulate type where the denticles and particles are present side by side [8]. According to Junquera et al. in 2005, three types of odontomas are recognized clinically, which include central (intraosseous) odontoma, peripheral (extraosseous or soft tissue) odontoma, and erupted odontoma [9].

Although the etiology is unknown, cases have been found to be related to local trauma, infection, from an exuberant proliferation of the dental lamina or its remnants [10]. Hitchin suggested that odontomas may be either inherited or due to a mutant gene or interference, possibly postnatal, with the genetic control of tooth development [11]. Herein we report a case of tooth erupting from the right buccal mucosa because of its rarity and the enigma in naming the lesion.

## 2. Case Report

**Figure 1:**
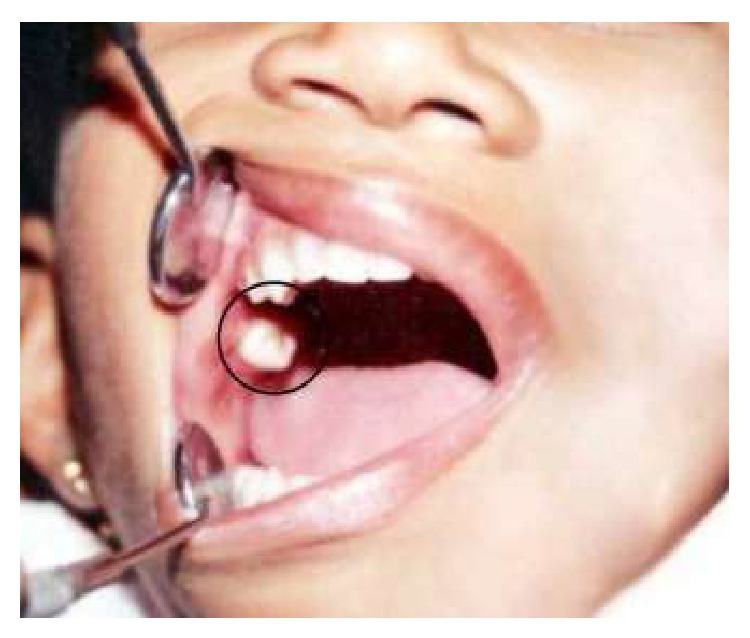
Intraoral view showing eruption of tooth-like mass from right buccal mucosa.

A six-year-old girl child was brought by her mother to the outpatient department with a chief complaint of tooth-like structure erupting from the right cheek region which started 2 months ago. The patient gave a history of mild pain and slight difficulty in mouth opening for the past two weeks. Her past medical and dental histories were noncontributory and she was otherwise a healthy child with a normal delivery. There was no prior history of trauma and no specific abnormality was noticed extraorally. On intraoral examination, a creamish-white, hard, 1 × 2 cm, molariform tooth-like structure was seen opposite to maxillary right first molar along the occlusal plane in the buccal mucosa (Figure 1). It was associated with mild inflammation and erythema of the surrounding mucosa and a provisional diagnosis of odontoma was given.

**Figure 2:**
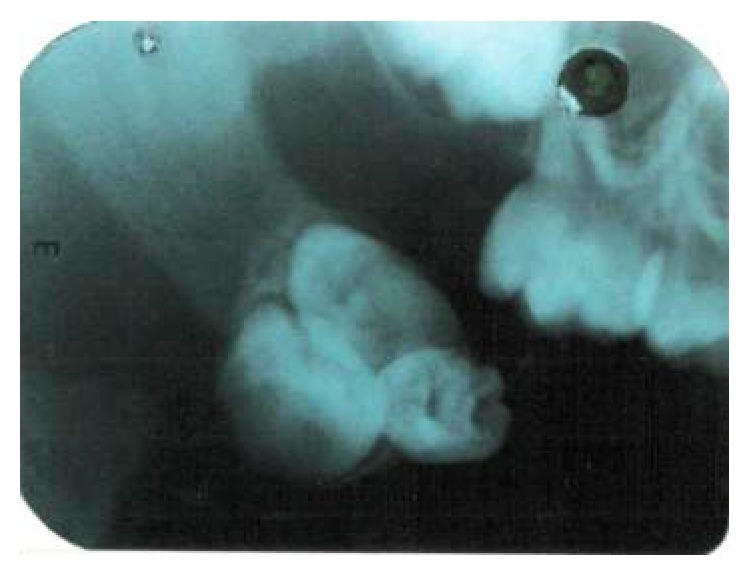
Intraoral periapical radiograph showing three radiopaque tooth-like masses with a radiolucent rim.

**Figure 3:**
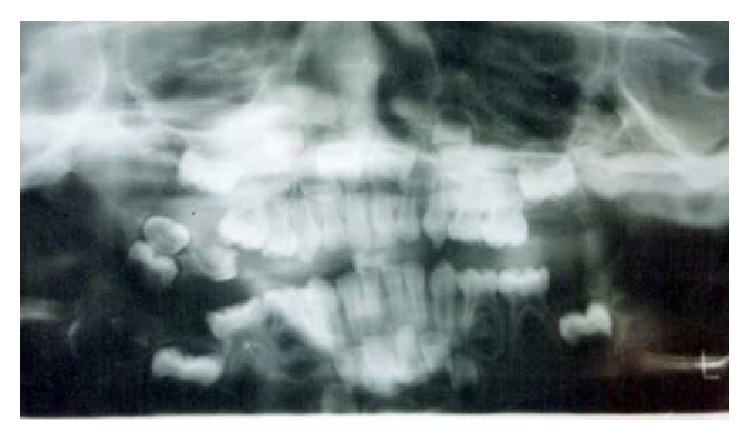
Orthopantomograph showing location of mass in the right buccal mucosa.

**Figure 4:**
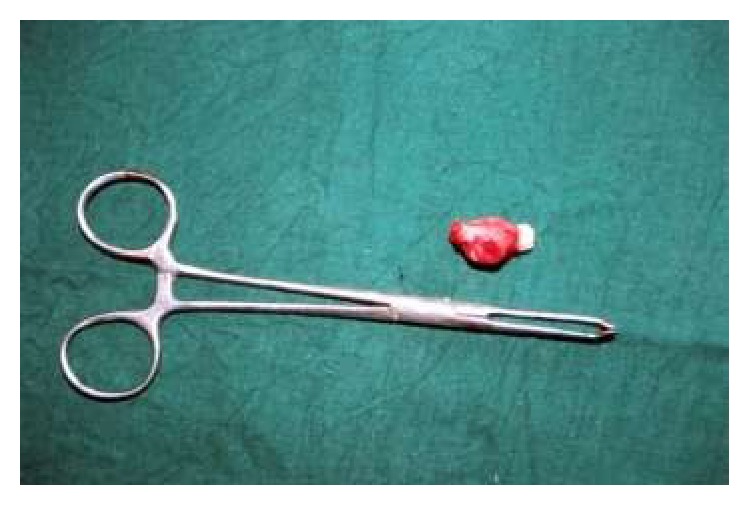
Macroscopic view showing tooth masses with attached soft tissue.

**Figure 5:**
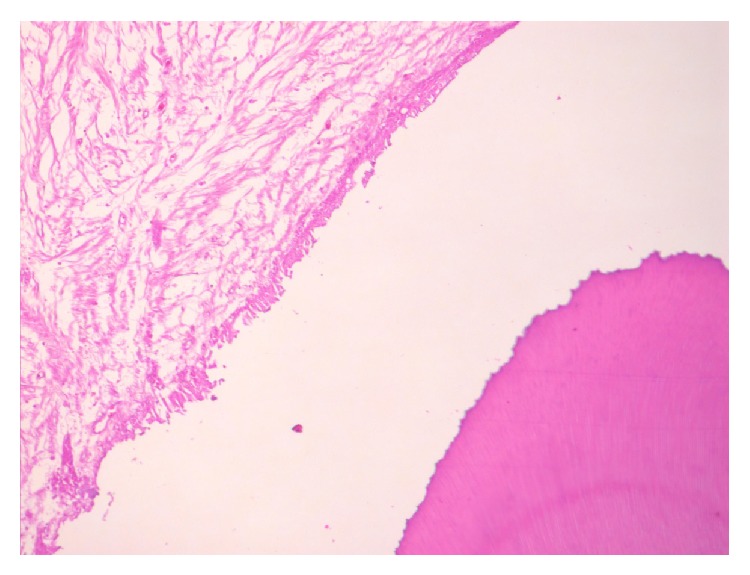
Histological section showing enamel space, scalloped dentinoenamel junction, dentinal tubules, and follicular tissue (objective 20x).

**Figure 6:**
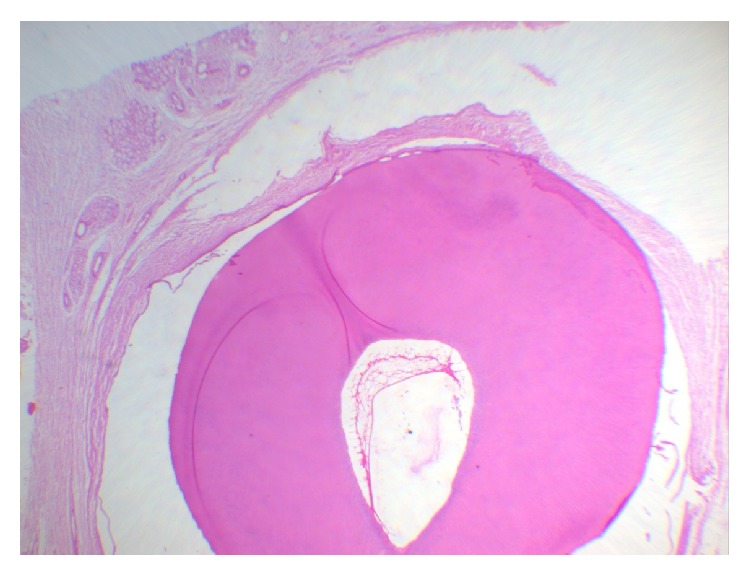
Histological section showing pulp space and fibrous connective tissue with minor salivary glands (objective 10x).

Radiologic examination revealed a radiopaque mass consisting of three tooth-like structures surrounded by a narrow radiolucent rim exclusively located in the posterior aspect of right buccal mucosa and no other ossifications were evident (Figures 2 and 3). Surgical excision of the mass was performed under local anaesthesia and the specimen was sent for histopathologic examination. Macroscopic examination revealed three miniature tooth-like structures with attached soft tissue (Figure 4). Microscopic examination of the decalcified section revealed an enamel space, dentinoenamel junction, dentin, pulp, and cementum in a manner similar to normal tooth (Figure 5). Fibrous connective tissue with islands of mucous minor salivary gland tissue owing to its location in the buccal mucosa was also evident (Figure 6). Based on all the above findings, a final diagnosis of compound odontoma was given.

## 3. Discussion

Odontoma by definition refers to any tumour of odontogenic origin; however, it means a growth where both the epithelial and mesenchymal cells exhibit complete differentiation [12]. Odontomas occur at any age but are most commonly seen in the first two decades of life which accords with the present case which had occurred in a six-year-old child. They are usually asymptomatic and are often detected on routine radiographic examination or when the permanent tooth fails to erupt. Most of the cases are intrabony lesions and very rarely they erupt spontaneously into the oral cavity [13, 14]. Erupted odontomas are associated with pain, inflammation, and restriction of mouth opening. In the present case also, one of the tooth-like structures had erupted from the right buccal mucosa and was associated with pain and difficulty in mouth opening. The first case of an erupted odontoma was described in 1980 by Rumel et al. [15].

Numerous reports have been reported where teeth are located in unusual locations [16] with most of the cases either partly or completely buried in the bone [17]. However, their location completely within the soft tissue remote from the jaws like upper lip, eyelid, and cheek is rare. They are being reported under diverse nomenclature such as irregular eruption, ectopic tooth, ectopic soft-tissue mesiodens, and ectopic odontoma [18]. The term ectopia is used to designate a condition where a tooth or odontoma is located or erupts in a place remote from the normal site. The present case showed eruption of tooth-like mass from the right buccal mucosa; hence the term ectopic can be used.

A search of literature revealed only three cases with eruption of tooth from the cheek region. The first case of odontoma in cheek was reported by El sefdy Bakry in 1977 in a 9-year-old girl with Klippel-Feil syndrome [17]. A second case of molariform tooth-like structure with a significant bony-stalk erupting from the cheek was reported by Noroozi and Arora in a nonsyndromic 2-year-old patient, who suggested the term “odontogenic choristoma” to the osseous tissue [19]. However, Ide and Mishima [20] questioned the suitability of this nomenclature. Ide et al. [21] opined that buccal mucosa is only an extragingival location in which a variety of odontogenic lesions can be encountered and also is a site of aborted odontogenesis under exceptional conditions. This is because of the common origin of dental and vestibular laminae. The authors also opined that cranially directed neural crest cells give rise to odontoblasts, cementoblasts, periodontal ligament fibroblasts, and osteoblasts during a sequential process of tooth development and suggested terming 1 or 2 recognizable teeth with no heterologous element of endodermal origin as* “supernumerary or accessory teeth”* if normal dentition is confirmed. The third case was by Liu et al. in a 4-year-old nonsyndromic girl child who found 2 mature molariform teeth in left buccal mucosa which was similar to the our case [22]. The authors Liu et al. [22] also agreed with the opinion of Ide and Mishima [20].

In the present case, the orthopantomograph of the child revealed the entire primary dentition and the developing permanent molar, incisor, and canine tooth buds. No bony relation of the molariform tooth was noted. The radiolucent rim seen is due to the follicular tissue [23] which was also confirmed with the histologic findings. Based on the available literature and excluding the history of trauma, the probable reasons for the occurrence of this mass include (i) fragmentation of the dental lamina during odontogenesis; (ii) pluripotent neural crest cells with latent odontogenic potential located in the buccal mucosa; (iii) genetic influence; (iv) a supernumerary tooth bud displaced to the extragingival location due to lack of space within the developing alveolar process before ossification of jaws has started (a rare probability).

In our case, no evidence of osseous tissue or cartilage is seen. So, the term choristoma cannot be applied in the current scenario. Though “supernumerary” is the term suggested by various authors, we found three tooth-like structures (denticles) attached together rather than a fully formed separate tooth structure which was confirmed both macroscopically and microscopically. Henceforth, after careful evaluation and compiling all the terms, we confer the present case as ectopic compound odontoma of denticular type.
